# The Interplay of Climate Change and Air Pollution on Health

**DOI:** 10.1007/s40572-017-0168-6

**Published:** 2017-10-28

**Authors:** H. Orru, K. L. Ebi, B. Forsberg

**Affiliations:** 1 Department of Family Medicine and Public Health, University of Tartu, Ravila 19, 50411 Tartu, Estonia; 2 Department of Public Health and Clinical Medicine, Umea University, Umea, Sweden; 3 Center for Health and the Global Environment, University of Washington, Seattle, WA USA

**Keywords:** Climate scenarios, Ozone, Fine particles, Emission, Dispersion, Uncertainty

## Abstract

**Purpose of Review:**

Air pollution significantly affects health, causing up to 7 million premature deaths annually with an even larger number of hospitalizations and days of sick leave. Climate change could alter the dispersion of primary pollutants, particularly particulate matter, and intensify the formation of secondary pollutants, such as near-surface ozone. The purpose of the review is to evaluate the recent evidence on the impacts of climate change on air pollution and air pollution-related health impacts and identify knowledge gaps for future research.

**Recent Findings:**

Several studies modelled future ozone and particulate matter concentrations and calculated the resulting health impacts under different climate scenarios. Due to climate change, ozone- and fine particle-related mortalities are expected to increase in most studies; however, results differ by region, assumed climate change scenario and other factors such as population and background emissions.

**Summary:**

This review explores the relationships between climate change, air pollution and air pollution-related health impacts. The results highly depend on the climate change scenario used and on projections of future air pollution emissions, with relatively high uncertainty. Studies primarily focused on mortality; projections on the effects on morbidity are needed.

## Introduction

Climate is an important factor that influences air quality. Pollutant emission, transport, dispersion, chemical transformation and deposition can be influenced by meteorological variables such as temperature, humidity, wind characteristics and vertical mixing [1]. In general, climate change is expected to worsen air quality in several densely populated regions by changing atmospheric ventilation and dilution, precipitation and other removal processes and atmospheric chemistry [2]. Reduced air quality will directly affect human health and will affect ecosystems in ways that also could affect human health and impact climate in a feedback loop [3, 4].

Several studies indicate that climate change has already affected air quality. For instance, Fang et al. [5] simulated that from pre-industrial (1860) to present (2000), the global population-weighted fine particle (PM_2.5_) concentrations increased by 5% and near-surface ozone concentrations by 2% due to climate change. According to Silva et al. [6•], the change from pre-industrial resulted in up to 111,000 and 21,400 additional premature fine particle- and ozone-related deaths due to climate change, respectively. Over the last two decades, roughly every degree of warming (°F) in the observed data was associated with an increase of 1.2 ppb in ozone concentrations [7]. As the climate continues to change, these impacts are projected to continue in the future.

The purposes of this review are to describe the interactions between climate, air pollution and health, to evaluate the recent projected impacts of climate on air pollution-related health impacts, to discuss the knowledge gaps and uncertainties and to identify research priorities for future study. We focus on the last 5 years, as earlier periods were well-documented in other reviews, e.g. [8–10]. Also, we concentrate on PM_2.5_ and tropospheric ozone, where most studies focussed.

## Interactions Between Climate, Air Pollution and Health

**Fig. 1:**
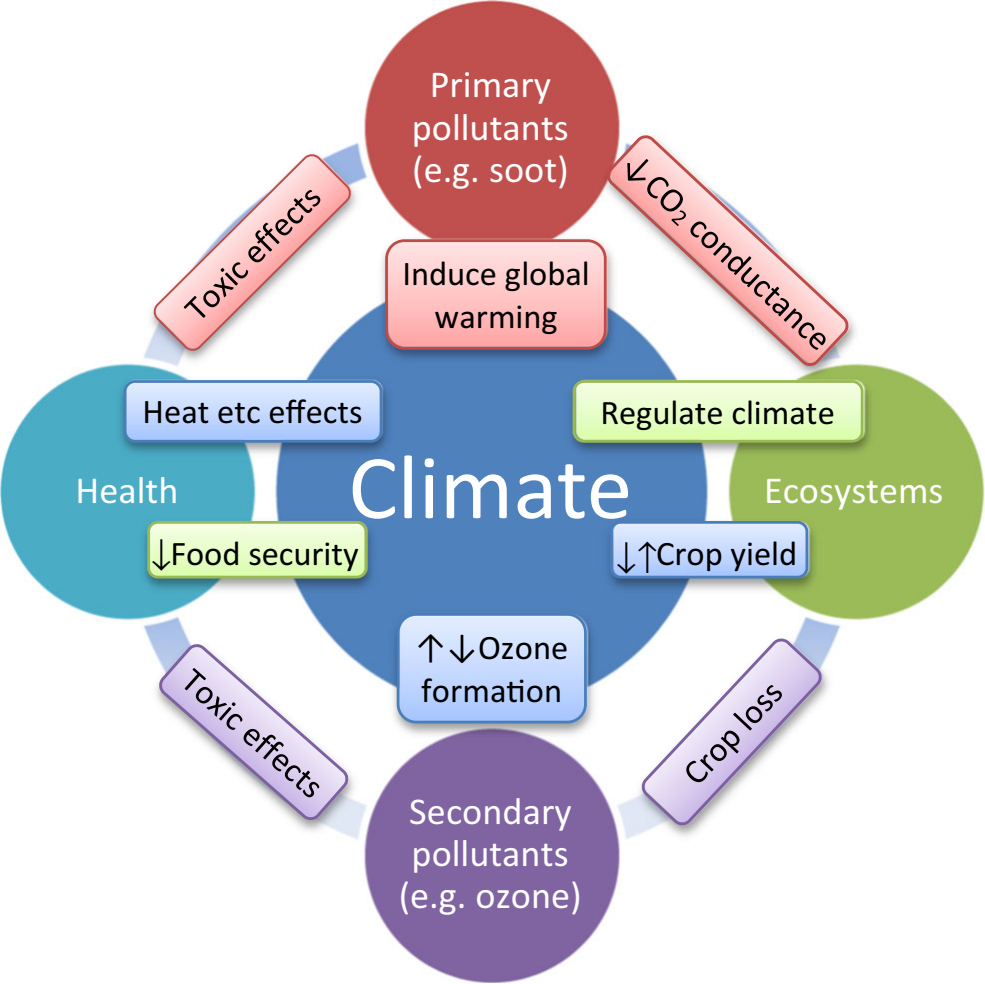
Climate and air quality interactions and direct and indirect effects on health

Climate can affect air quality, air quality can affect climate change and both can directly or indirectly affect health (Fig. 1). The two major effects of climate change on air quality are degrading the removal processes (dispersion, precipitation) and amplifying the atmospheric chemistry [2]. These will affect primary (e.g. soot particles) and secondary (e.g. ozone and sulphate particles) pollutants.

Higher concentrations of PM_2.5_ from anthropogenic sources can result from changes in precursor emissions, in meteorology and in the physical and chemical behaviour of particles in the atmosphere [11•]. Besides anthropogenic emissions, future climate will change the emissions of biogenic volatile organic compounds (BVOCs) due to higher temperatures and changed plant metabolism; this can alter secondary organic aerosols (SOAs) resulting in changes in secondary particle levels [12]. More and larger wildfires associated with climate change could significantly reduce air quality by the end of the century [13]. Other natural sources include dust storms and dust particle transport; climate change could increase their frequency [11•]. For example, Jacob and Winner [14] concluded that due to climate change (mainly due to weaker global circulation and a decreasing frequency of mid-latitude cyclones), annual mean PM_2.5_ concentrations will change by ± 1 μg/m^3^ in the USA and Europe.

PM_2.5_ also can affect the climate. Soot (black carbon) can absorb heat, thus increasing local temperatures [15]. Other secondary particulates, such as sulphate particles, cool the climate and contribute to aerosol–cloud interactions [16, 17]. This includes the potential for mitigation of climate change by reducing soot levels as well as by engineered cooling particles [15, 18].

Near-surface ozone is another secondary pollutant formed from the interaction of precursor compounds with sunlight, including UV radiation [14]. The rate of formation is temperature-dependent. Because of this, sunny and cloudless days and higher temperatures are more conducive to higher ozone concentrations. Wind can control ozone levels by dispersing precursor species, thus reducing ozone formation. Dry deposition (to vegetation, surfaces) also removes ground-level ozone [2]. The formation of near-surface ozone is the result of chemical reactions that depend on ozone precursor emissions from natural and anthropogenic sources. The main precursors include several primary and other secondary pollutants such as VOCs, CH_4_ and CO that react with hydroxyl radical (OH) to ultimately produce ground-level ozone (Fig. 1). Increasing temperatures due to climate change generally lead to increased natural VOC emissions that affect ozone concentrations [19]. Further, hydroxyl radical formation is associated with methane, another greenhouse gas [20].

The interactions between air quality and health are direct and indirect (Fig. 1). First, particles, especially from combustion, can affect cardiopulmonary mortality, hospitalization and respiratory disease (e.g. asthma, chronic bronchitis, rhinitis) [3]. Recent evidence supports associations with diabetes [21], rheumatic diseases [22], cognitive functioning [23] and neurodegenerative diseases [24]. Furthermore, gases such as the secondary pollutant ozone are related to all-cause, circulatory and respiratory mortalities [3, 25] as well as chronic respiratory diseases such as asthma [3]. Studies have connected higher ozone concentrations with preterm birth [26], reproductive health [27] and cognitive decline [24].

Second, primary and secondary pollutants can boost climate change that in turn affects public health through, for example, more extreme temperatures [28]. Secondary pollutants such as ozone can also affect crop yields that, in combination with climate, can affect food security and public health [29, 30]. Tai et al. [31•] concluded that climate change could reduce global crop production by > 10% by 2050. Therefore, climate change could significantly indirectly affect public health, especially in poorer countries.

## Projected Impacts of Climate Change on Air Pollution-Related Health Effects

Quantifications of the impacts of climate on air pollution-related health effects use (1) future air pollution concentrations, (2) current and/or future population and mortality data and (3) concentration–response functions between air pollution exposure and mortality/morbidity from earlier epidemiological studies. The future air pollution models used input from global climate models that were downscaled to drive a regional, numerical model in higher spatial resolution, to simulate local conditions in greater detail. Global climate models apply different greenhouse gas emission scenarios that modify the magnitude and pattern of climate change (e.g. global temperature increase) in different projections. Air pollution models use data on meteorology, dispersion, chemistry and deposition, projections of future climate and primary pollutant emissions. Based on future air pollutant exposures and mortality/morbidity rates, the future impacts of climate change on air pollution-related health effects can be evaluated.

We limited our review to studies where the effect of climate change on air quality was projected and the change in health effects was quantified. A literature search was conducted in April 2017 using electronic databases Pubmed, Web of Science, ScienceDirect and Scopus. We focused only on peer-reviewed journal articles published in English from 2012 to 2017. We used key words ‘Climate Change’, ‘Fine Particles’, or ‘Ozone’ and ‘Health’ and also looked for studies cited in the identified articles. The search identified 17 studies that were selected for the review (Table 1).

**Table 1:** Projected impacts of climate change (CC) on fine particle (PM_2.5_) and ozone (O_3_) related health effects and characteristics of studies published in 2012-2017

Region	Calculated health effects	Used CC projection scenario	Air pollution emissions and dynamics	Used CC and air pollution models	Included periods	Additional factors taken into account	Main results	Reference
Global	PM_2.5_ related all-cause and O_3_ related respiratory mortality	A1B	Present constant	GFDL, AM3	1981-2000, 2081-2100	-	In 2081-2100 4% increase in PM_2.5_ related all-cause mortality and 0.9% in O_3_ related respiratory mortality compared to 1981-2000	[30]
Global and different regions	PM_2.5_ and O_3_ related mortality	RCP2.6, RCP4.5, RCP6.0, RCP8.5	Natural dynamic, anthropogenic constant	ACCMIP model ensemble	2000, 2030, 2050, 2100	Population projection from 2010 through 2100	PM_2.5_ related mortality peaking in 2030 (2.4-2.6 million deaths annually and then declining to between 0.56-1.55 million deaths annually (except for RCP6.0) O_3_ related mortality peaking in 2050 (1.18-2.6 million deaths annually) and then declining to between 1.1-2.4 million deaths annually	[31]
Global, Europe, France	PM_2.5_ related cardiovascular (CV) and O_3_ related respiratory mortality	Only for Europe and France (RCP4.5)	Present and future (CLE, MFR)	IPSL-cm5-MR, LDMz-INCA, CHIMERE	2010, 2030, 2050	Population 2030 as sensitivity analysis	In 2030 in Europe PM_2.5_ related CV mortality decrease by 1.9% under CLE and 2.2% under MFR and in 2050 3.8% decrease under both scenarios compared to 2010. In 2030 O_3_ related respiratory mortality decrease by 0.2% under CLE and 0.3% under MFR compared to 2010	[29]
Europe	O_3_ related non-accidental mortality and respiratory hospitalizations	A1B, A2	Present constant	MATCH-RCA3, ECHAM4, HadCM3	1961-1990, 1990-2009, 2021-2050, 2041-2060	-	In 2021-2050 13.7% increase in O_3_ related non-accidental mortality with A2 scenario and 8.6% increase with A1B scenario compared to 1961-1990	[33]
Europe	O_3_ and PM_2.5_ non-accidental mortality	A1B	Present and future (in accordance to RCP4.5)	ECHAM5, DEHM, MATCH	2000s, 2050s, 2080s	Population projection 2050, PM_2.5_ infiltration change in the future	Climate only: in 2050s 8-11% increase and in 2080s 15-16% increase in non-accidental mortality compared to 2000s (O_3_ and PM_2.5_ combined) Climate and emissions combined: in 2050s 36-64% and in 2080s 53-84% decrease in O_3_ related non-accidental mortality compared to 2000s and in 2050s 62-65% and in 2080s 78-79% decrease in PM_2.5_ related mortality compared to 2000s	[34]
UK	O_3_ non-accidental mortality and morbidity	A2, B2	Present and future (CLE, MFR)	EMEP-WRF	2003, 2030	Population projections, +5 °C temperature increase scenario	If temperature will increase by 5 °C, the O_3_ related non-accidental mortality will increase from 4% (no O_3_ threshold) to 30% (35 ppbv O_3_ threshold)	[36]
Poland	PM_2.5_ non-accidental mortality	A1B	Present constant	ECHAM5-RegCM3, CAMx	1990s, 2040s, 2090s	-	Compared to 1990s 6% decrease in PM_2.5_ related non-accidental mortality in the 2040s and 7% decrease in 2090s	[37]
US	O3 summer-time non-accidental mortality	A1B, A2, A1Fi, B1	Biogenic and evaporative dynamic, anthropogenic constant	2 global and 5 regional modelling systems	2000, 2050	Population projection 2050	In 2050 depending on model choice from 600 avoided premature deaths due to O_3_ to 2,500 additional premature deaths compared to 2050	[42]
US	O_3_ related mortality and morbidity	A1, B2	Present and future (EPA, 2030)	GISS and CESM, WRF, CMAQ	1995-2005, 2025-2035	-	In 2030s annually 37 and 420 additional excess deaths due to O_3_ compared to 2000s with RCP6 and 8.5 scenarios, respectively	[41]
US	PM_2.5_ and O_3_ related annual mortality	RCP8.5	Present and future (in accordance to RCP8.5)	CESM, WRF, CMAQ	2002-2004, 2057-2059	Population projection 2050	In 2050s 7,500 additional PM_2.5_ related and 2,100 additional O_3_ related premature deaths with population kept constant and 42,600 less PM_2.5_ related and 1,300 additional O_3_ related premature deaths with 2050s population	[43]
US	PM_2.5_ related annual and O_3_ related summer-time mortality	POL4.5, POL3.7	Present constant	IGSM-CAM, CAM-Chem	1980-2010, 2035-2055, 2085-2115	-	In 2050 11,000 and 13,000 and in 2100 52,000 and 57,000 avoided PM_2.5_ and O_3_ related premature deaths compared to 2000, respectively for POL4.5 and POL3.7 scenario	[39]
US	O_3_ summer-time non-accidental mortality	A2	Present and future (in accordance to RCP8.5)	Global and regional climate and ozone models +Bayesian model	2000, 2050	-	In 2050 1,212 additional O_3_ related premature mortalities with present emissions and 4,473 less premature mortalities with future emissions compared to 2000	[48]
94 US urban areas	O_3_ summer-time non-accidental mortality	RCP6.0	Biogenic dynamic, anthropogenic constant	Spatial monotone ozone-temperature surface model	1995-2005, 2025-2035	Both 2000 and 2030 population	In 2025-2035 from 7.7% (35 ppb O_3_ threshold) to 14.2% (75 ppb O_3_ threshold) increase in O_3_ related non-accidental mortality compared to 1995-2005	[49]
Atlanta metropolitan area	O_3_ related ED visits	A2	Present and future (OECD90)	8 different models	1999-2004, 2041-2070	-	In 2041-2070 annually from 267 to 466 (depending on model) excess O_3_ related ED visits compared to 1999-2004	[53]
Japan	PM_2.5_ related mortality	RCP4.5	MIROC-AOGCM	NICAM-Chem, high and low resolution model	2000-2003, 2030-2033	Population projection 2030	In 2030s from 63.6% increase (0 μg/m^3^ threshold) to 8.7% decrease (5.8 μg/m^3^ threshold) in PM_2.5_ related mortality compared to 2000s (high resolution model)	[46]
Korea	O_3_ summer-time non-accidental mortality	RCP2.6, RCP4.5, RCP6.0, RCP8.5	Present and future (in accordance to RCPs)	ICAMS	1996-2005, 2016-2025, 2046-2055	Current mortality trends expected to increase, temperature effects compared	In 2020s from 0.0% to 0.5% increase and in 2050s from 0.2% to 0.6% increase in O_3_ related non-accidental mortality compared to 2000s	[48]
Sydney	O_3_ related mortality	A2	Present constant	CGCM, CCAM, TAPM-CMT	1996-2005, 2051-2060	-	In 2050s from 27.3% (40 ppb O_3_ threshold) to 2.3% (0 ppb O_3_ threshold) increase in O_3_ related mortality compared to 2000s	[47]

The majority of studies focused on ozone, but compared with earlier analyses, there was a higher proportion of studies on PM_2.5_ [8, 9]. Six studies included analyses of the health impacts of both. The 17 studies covered a wider geographic range than earlier studies.

### Global Projections

Three studies over the last 5 years projected globally how climate change could affect mortality; all included analyses of PM_2.5_- and ozone-related mortalities (Table 1). One study conducted by Likhvar et al. [32] included all of the world in current climate assessment, but included future climate projections only for France and for all of Europe, with global emission change taken into account. Fang et al. [33•] also analysed one climate change projection, while Silva et al. [34] used four. Using more than one projection of climate change provides insights into the range of uncertainties about future air quality and associated health impacts. Overall, climate is expected to increase PM_2.5_- and ozone-related mortalities [33•], peaking in 2030 and 2050, respectively, followed by a decrease due to reductions in emissions [34] that outweigh the effects of climate change. The measures reducing emissions include adopting and enforcing next-generation standards and policies (more stringent than Euro 6/VI) that could reduce significantly premature mortality [35]. Larger climate change effects are expected globally (especially in Asia) due to projected increases in PM_2.5_, with less impact of climate change on ozone-related mortality; Fang et al. [33•] projected a 4 and 0.9% increase in mortality associated with these pollutants by the end of the century, respectively.

### European Projections

Two studies focused on the whole of Europe [36, 37]. In addition, in one global study [32], Europe was one of the included regions; however, the effects of climate change were combined with effects from emission change (Table 1). In their projections, Orru et al. [36] projected climate effects only on ozone-related mortality, keeping all other factors constant. Larger effects were observed with higher greenhouse gas emissions (the A2 scenario) compared to lower greenhouse gas emissions (A1B), with much larger effects expected by the middle of the century compared to today. The A2 scenario represents a very heterogeneous world with high population growth and relatively rapid economic development based on fossil fuels, whereas A1B reflects introductions of new and more efficient technologies after rapid economic growth [38]. There also were large regional differences under these two scenarios: a large increase was projected in central and southern Europe, whereas decreasing effects are expected in northern Europe. Geels et al. [37] also included changes in emissions, population and PM_2.5_ infiltration (future tighter buildings) in their modelled scenarios and assessments. Their projections suggested up to two times larger effects of climate at the end of the century compared to the 2050s. Changes in emissions would outweigh the effects, with large decreases (36–84%) projected in ozone- and PM_2.5_- related mortality.

Besides European-wide studies, there were three national-level analyses (Table 1). A study in the UK projected that a 5 °C temperature increase with future projected emissions of ozone precursors could increase ozone-related mortality by 4–30% depending on the assumed threshold for the health impacts of ozone [39]. A study in Poland projected PM_2.5_- related mortality, keeping all factors constant except climate [40]. Under that assumption, climate change may lower the levels of anthropogenic PM_2.5_ in future decades presumably due to increased winter precipitation [41], thus reducing air pollution-related mortality. The global study that included France projected a delay in around 13,000 annual cardiovascular deaths in 2050 compared to 2010, with larger reductions in mortality in the city area of Paris [32].

### US Projections

Five studies recently projected health risks for the entire USA; one study included a large number of US urban areas and one the Atlanta metropolitan area only (Table 1). Five studies focused on ozone and two on ozone and PM_2.5_. In general, all studies projected increases in ozone- and PM_2.5_-related mortalities due to climate change. Among the studies, Garcia-Menendez et al. [42] was different because two policy scenarios were compared, and both projections indicated a large number of avoided deaths compared to a no-policy scenario. The number of avoided deaths increased with a stricter greenhouse gas scenario (3.7 vs 4.5 W/m^2^). Other studies frequently used the A2 emission scenario, similar to the currently more widely applied representative concentration pathway (RCP) 8.5, finding larger health risks of climate change than under lower emission scenarios (Table 1). RCPs are four greenhouse gas emission trajectories resulting in specific levels of radiative forcing in 2100; these reflect the possible range of radiative forcing values in the year 2100 relative to pre-industrial values (+ 2.6, + 4.5, + 6.0 and +8.5 W/m^2^, respectively) [43]. IPCC [44] has defined radiative forcing as the change in net (down minus up) irradiance (solar plus longwave; in W/m^2^) at the tropopause after allowing for stratospheric temperatures to readjust to radiative equilibrium, but with surface and tropospheric temperatures and states held fixed at the unperturbed values.

In other studies, Fann et al. [45] have projected the health risks until the 2030s to be smaller than those projected to be experienced in the middle of the century [46, 47], with even larger risks by the end of the century [42]. Climate change will increase exposures to higher concentrations of ground-level ozone; the magnitude of risks will depend on the threshold assumed (e.g. if we expect ozone health effects starting at 70 ppb as the National Ambient Air Quality Standard in the USA compared with the 35 ppb that has been often used in health impact assessments) [48]. As with projections from Europe, the effects of emission change are expected to outweigh the effects of climate change, resulting in smaller air pollution-related health burdens in the future [49].

### Asian and Australian Projections

Our literature search identified two projections from Asia (in addition to global projections) and one study from Sydney, Australia (Table 1). All studies projected that climate change would increase air pollution-related mortality; the results depended on the climate change projection and pollutant effect threshold. For example, Goto et al. [50] from Japan showed climate to increase PM_2.5_-related health risks when assuming the no-health-effect threshold. The projected risks decreased when assuming a threshold of 5.8 μg/m^3^, below which no health effects are expected. For ozone, the threshold assumed affects the projected risks, as shown in the Physick et al. [51] study that projected larger increases in ozone-related mortality at higher threshold levels (40 ppb). A Korean study confirmed earlier results from other regions that increased emissions under the RCPs associated with higher radiative forcing would result in greater health risks [52•]. Also, up to a 0.6% increase in ozone-related mortality by the 2050s was projected despite decreased concentrations of ozone precursor emissions; this differs from projections from Europe and the USA [32, 37, 49].

## Discussion

Over the last 5 to 6 years, 17 studies projecting the health risks of climate change on air pollution health effects were identified. Since earlier reviews, additional studies were published concerning the health risks of projected future concentrations of ozone and PM_2.5_, with a proportionally larger increase in publications on PM_2.5_ [8–10]. All studies projected climate change to increase air pollution-related health effects, but in several studies, the projected health effects of air pollution due to climate change alone were small compared with the effects associated with the health effects from direct emissions of those pollutants.

One of the primary challenges in this literature is that all of the results are projected under unknown future scenarios. Thus, there are inherent uncertainties related to future emissions and the resulting climate change that will impact the projected air pollution-related health impacts. In addition, there is often little consistency across studies in terms of assumptions made. For example, almost all the studies evaluated use different scenarios of climate change, air pollution emissions, time periods of analysis and baseline data (either static or dynamic). This makes clear that the comparison of the studies is nearly impossible. While it is certainly important to take sensitivities (emission, population change, etc.) into account, it would be crucial to have also projections of the air pollution-related health risks of climate change alone, with subsequent analyses providing additional information on the major sensitivities and uncertainties.

In the following sections, we discuss current uncertainties in the climate change projections, air pollution emissions, climate change and air pollution models and the health impact assessment data.

### Future Climate Change Projections

The studies used a large number of different possible future greenhouse gas emission scenarios to project the future climate and air pollution, with A1B and RCP4.5, which assume a moderate change, being the most common. These scenarios describe possible future emissions of greenhouse gas emission and often air pollutants also. There is obviously high uncertainty about future development pathways and resultant emissions. In addition, anthropogenic greenhouse gas emission-driven climate change is another source of uncertainty for projections of air pollution [53]. Using multiple scenarios is becoming the best practice for indicating the range of uncertainties in projections [34, 36, 39, 42, 45, 46, 52•]. Using this approach, it appears that until the 2050s, differences between emission scenarios are rather small because of the inertia in the climate system [10] but emission scenarios become increasingly divergent by the end of the century [34].

### Air Pollution Emissions

Studies typically kept air pollution or anthropogenic emissions constant or projected future emissions. Where future emissions were projected to decrease, significant decreases in pollution levels were expected [37]. However, even under these conditions, studies did not project such a big decrease for Europe [32] and in some areas, climate change might increase the air pollution-related health burdens due to aging population which increases the proportion of vulnerable persons and/or migration, even as air pollution emissions decrease [52•, 54]. Additionally, decreases in future emission can be lower than those that the earlier studies projected because currently there have been disagreements between current emission inventory estimates and earlier projections [55]. For example, under the SSP3 and SSP4 (shared socioeconomic pathway) scenarios, economic, institutional and technological limitations slow air quality improvements [56] such that projected global pollutant emissions over the twenty-first century may be comparable to current levels [57]. Nevertheless, the air pollution models are highly sensitive to the input emission data and if the real future emission would not follow the projected trends, the hereafter conclusions could be significantly different.

### Climate Change and Air Pollution Models

The majority of studies applied different air chemistry–transport models. Two studies also applied statistical models. Alexeeff et al. [49] applied Bayesian modelling and Wilson et al. [48] spatial monotone modelling. The spatial coverage of chemistry–transport is in general better, but still rather crude, often 0.5°, due to large computational resources needed. In general, the magnitude of effects in chemistry–transport and statistical models is similar, but different climate change projections, time periods and baseline data were applied, meaning it is very difficult to compare the results in detail.

The choice of the climate change model used in the projections could be significant, with increasing or even decreasing effects expected [46, 54]. This means that models could under- or overestimate the actual effects. The main uncertainties could rise from model parameters and/or interactions between climate and air quality, often characteristic to a certain region [44]. To address these uncertainties, an ensemble of models and a downscaling technique should be applied. But as was shown [9, 10] and in Table 1, often, a single global climate projection was used, probably due to the accessibility and capacity of computational resources.

### Health Impact Assessment and Baseline Data

The majority of the studies used health impact assessment concentration–response functions (CRFs) that were based on a multi-city study or meta-analysis of studies from several countries. Several of the ozone studies only applied CRFs based on short-term exposure effects, while the PM_2.5_ analyses only took long-term effects into account. Assuming short-term exposure effects only from ozone exposure probably underestimated the effects, although there are a few of long-term effect studies [58]. Newer research (e.g. [25]) concluded that there may be additional adverse health impacts of ozone. Results will depend on both the assumed CRF and the inclusion of a threshold assumptions or cutoff. Studies using CRFs typically assume a linear relationship, which may not adequately describe the association between ozone and health effects, as shown in the modelling by Wilson et al. [48] using non-linear, monotone, bivariate health impact functions. Based on what is known about the CRFs, if the concentrations are moderate, no significant difference should appear, but at very low or high pollutant levels, significant differences are possible [59].

Using the current CRFs, mortality rates and exposed population at baseline could also introduce errors into the projected air pollution-related health impacts of climate change because of demographic changes, socioeconomic development and other factors [60]. Several studies used future populations in future assessments, which provide more confidence in the projected risks (Table 1). For instance, Wilson et al. [48] projected significantly higher (39.8 vs 7.7) percent change in excess mortality attributable to ozone concentrations above 40 ppb if population changes by 2030 were taken into account. Not all studies used these projected estimates, however. Similarly, another source of uncertainty pertains to the timing of the mortality rates used given an overall decreasing trend in age-standardized death rate over recent decades, although the rate of decrease appears to have slowed for cardiovascular and other air pollution-related diseases during the last years in the USA [61]. Therefore, careful consideration should be made regarding the mortality rates adopted as following the earlier trends could overestimate future effects.

## Conclusions and Perspectives

Climate change is generally expected to increase air pollution concentrations in the future, although a decrease in pollutant emissions would reduce the negative effects of climate change in the future, leading to an improvement in projected air quality. Several studies projected larger relative increases in PM_2.5_- than in ozone-related health effects [5, 47]; PM_2.5_ is the primary pollutant contributing to the health burden of air pollution [62]. However, there was some evidence that in certain regions (e.g. in northern Europe and Poland), climate change could somewhat improve air quality, primarily due to change in long-range atmospheric air pollution transportation [36, 40]. As climate change impacts on air quality vary globally, more regional assessments are needed, especially in low- and middle-income countries that currently have higher air pollution concentrations. Taking consistent approaches across studies would facilitate comparisons of these regional assessments.

The review highlighted several key research gaps. The 17 analysed studies focused on the direct effects of air pollution on health, principally premature mortality. But the consequences of exposure to higher concentrations are much wider than premature mortality. Only five studies included morbidity effects, which mean that other health effects, for example, in children, are mostly left out. Better understanding also is needed of the indirect effects of air pollution on human health through crop loss and resulting malnutrition. These kinds of effects have yet been taken into account.

Another key research need for future studies is to compare the health burdens of future air quality under a changing climate with alterations in other risk factors due to climate change. For example, only one study jointly compared the effects of air pollution and temperature [52•]. Even if air pollution health risks might decrease because of emission reductions, ambient temperatures and heat waves are still expected to increase [63]. Further understanding is needed because exposure to higher temperatures and higher concentrations of air pollutants may have synergistic effects, especially on cardiovascular disease [64], causing even higher health burdens than exposure to each individually. Understanding where this could occur would inform regulations of emissions to help reduce future health burdens. Similarly, indoor air quality will likely be impacted by climate change via mechanisms like mould growth as climate change increases the probability of storms and flooding that can impact health [65].

Since the future health burdens of air pollutants, particularly PM_2.5_ and ozone, will depend on actions taken to reduce the responsible emissions, better understanding is needed of the emissions of air pollutants under multiple development pathways. Therefore, increased investment in understanding the range of possible future health risks of changes in air pollution under a range of future possible climates and emissions is critical to inform regulations. Because current emissions have exceeded the projected trends [55], reducing emissions requires even stricter regulatory control technologies than currently planned.

Recent publications confirm that climate change will likely increase the concentrations of near-surface ozone and particulate matter, with associated adverse health consequences and with large uncertainties. Because there are very limited options for reducing human vulnerability to air pollutants, protecting population health under future warmer climates will require regulatory interventions such as reducing greenhouse gas emissions. Research is needed to quantify the magnitude and pattern of future risks, considering the full range of morbidity and mortality, to inform these stricter regulations.
